# Multidrug-Resistant *Acinetobacter baumannii*: Risk Factors for Mortality in a Tertiary Care Teaching Hospital

**DOI:** 10.3390/tropicalmed10010015

**Published:** 2025-01-06

**Authors:** Kristina Černiauskienė, Astra Vitkauskienė

**Affiliations:** Department of Laboratory Medicine, Faculty of Medicine, Medical Academy, Lithuanian University of Health Science, Eivenių˛ Str. 2, LT-50161 Kaunas, Lithuania; astra.vitkauskiene@lsmuni.lt

**Keywords:** *Acinetobacter baumannii*, multidrug resistance, risk factors, mortality, comorbidity, hospitalization

## Abstract

Background and objectives: Due to resistance and the lack of treatment options, hospital-acquired *Acinetobacter baumannii* (*A. baumannii*) infections are associated with high mortality. This study aimed to analyze the characteristics of patients with infections caused by multidrug-resistant (MDR) *A. baumannii* and patients’ clinical outcomes as well as determine the risk factors for mortality in a tertiary care teaching hospital. Materials and methods: A retrospective cohort study including 196 adult patients with *A. baumannii* strains isolated from different clinical specimens in the Hospital of the Lithuanian University of Health Sciences in 2016, 2017, 2020, and 2021 was conducted. Data on patients’ characteristics, comorbid diseases, treatment, length of hospital and ICU stay, and outcome were collected. Carbapenemase-producing isolates were detected phenotypically. To determine risk factors for in-hospital mortality, logistic regression analysis was performed. Results: There were 60 (30.6%) women and 136 (69.4%) men with a mean age of 61.7 ± 16.6 years (range, 52–74). More than three-fourths (76.5%, *n* = 150) of the patients had at least one comorbid disease. The highest number of *A. baumannii* strains were isolated from patients hospitalized in ICUs (43.4%, *n* = 85). *A. baumannii* strains producing three types of *β*-lactamases were more frequently isolated from women than men (77.8% vs. 22.2%, *p* = 0.006). Infections caused by *A. baumannii* strains producing two types of *β*-lactamases were significantly more often treated with combination therapy than infections caused by strains producing one type of *β*-lactamase (78.9% vs. 60.0%, *p* = 0.019). Patients with *A. baumannii* strains producing two different types of *β*-lactamases (AmpC plus KPC, AmpC plus ESBL, or ESBL plus KPC) stayed significantly shorter at the ICU compared to patients with *A. baumannii* strains with no detected *β*-lactamases (median of 9, IQR 2–18, vs. median of 26, IQR 7–38, *p* = 0.022). Death occurred in 58.7% (*n* = 115) of patients. Logistic regression analysis showed that a duration of the effective antibiotic treatment of ≤6 days, invasive mechanical ventilation, combination therapy, aged >58 years, and the absence of co-infection were independent predictors of in-hospital mortality. Conclusions: MDR *A. baumannii* infections pose a significant threat to human health not only due to multidrug resistance but also due to high mortality. The mortality rate of patients with MDR *A. baumannii* infection was high and was associated with age, invasive mechanical ventilation, the duration of effective antibiotic treatment, no co-infection, and combination therapy. Therefore, it is of utmost importance to reduce the prevalence of MDR *A. baumannii* infections in healthcare facilities by applying preventive measures and to administer timely effective treatment once *A. baumannii* infection is detected.

## 1. Introduction

Nowadays, *Acinetobacter baumannii* (*A. baumannii*) has been recognized as one of the major troublesome pathogens, capable of surviving in hospital environments and being responsible for healthcare-associated infections (HAIs) worldwide. It is known to be associated with several types of HAIs including invasive infections such as pneumonia, bacteremia, and osteomyelitis, as well as skin and soft tissue infections. With the emergence of HAIs, *A. baumannii* has become an important cause of mortality in critically ill patients [1]. Moreover, *A. baumannii* has been linked to community-acquired infections, although to a lesser extent [2,3]. Evidence of multidrug-resistant (MDR) *A. baumannii* is constantly accumulating, especially in intensive care units (ICUs), where it causes high mortality in critically ill patients [4]. Moreover, the overwhelming majority of ICU patients are given combined broad-spectrum antimicrobial agents at large quantities, leading to weaker immunity and increased susceptibility to hospital infections [5]. Infections caused by *A. baumannii* are increasingly difficult to treat due to the emergence of carbapenem-resistant and MDR *A. baumannii* strains [6]. Antibiotic resistance is associated with the production of β-lactamases: AmpC β-lactamases play an important role in resistance to β-lactam antibiotics, extended-spectrum β-lactamases (ESBLs) confer resistance to broad-spectrum cephalosporins, and *Klebsiella pneumoniae* carbapenemase (KPC) is associated with carbapenem resistance [7,8,9].

Thus, currently, there is no consensus on the strategies for the optimal treatment of carbapenem-resistant *A. baumannii* infections, and different guidelines advocate different recommendations and advice [10,11]. Obviously, carbapenems cannot be prescribed empirically where the resistance of *A. baumannii* to carbapenems is high [12]. Recently, polymyxins (cationic lipopeptides) are antimicrobial agents possessing the greatest bactericidal activity in vitro against *A. baumannii* (usually combined with other molecules such as meropenem, sulbactam, tigecycline, or fosfomycin). However, their irrational use can lead to further antibiotic resistance, and their clinical utility is limited by neurotoxicity and nephrotoxicity [13].

The updated Bacterial Priority Pathogen List (BPPL) 2024 published by the World Health Organization (WHO) includes carbapenem-resistant *A. baumannii* as a critical priority pathogen, which is resistant to many antibiotics and poses a threat to human health. The development of new and necessary treatments to fight infections caused by these pathogens and to stop the spread of antimicrobial resistance is of crucial importance [14]. Cefiderocol is a new siderophore cephalosporin, for which in vitro activity against MDR Gram-negative bacteria has been reported [15]. However, since no results of large and homogenous, randomized clinical trials are available, additional clinical data on the use of cefiderocol in severe carbapenem-resistant *A. baumannii* infections are urgently needed to definitively assess its efficacy and reach a consensus among the different guidelines [16].

MDR *A. baumannii*-caused infections pose challenges to early diagnosis and treatment, resulting in longer hospital stays and rising rates [2,3]. Age, chronic comorbid conditions, bedridden status, venous catheterization, ICU stay, infections with MDR phenotypes, and concurrent fungal infections have been recognized as risk factors for *A. baumannii* infection-related mortality in many studies [17,18]. In the study by Muntean et al. [19], previous antibiotic therapy at admission, blood transfusion, and ulcer pressure have been identified as risk factors for the development of *A. baumannii* infection in patients admitted to the ICU. The study by Abarca-Coloma et al. [20] showed that based on the multivariate Cox regression analysis, the main risk factors associated with mortality were a history of chronic renal failure, hemodialysis, and invasive mechanical ventilation (IMV) exposure. With the findings from different studies being inconsistent, it is of crucial importance to analyze risk factors for MDR *A. baumannii* infections in patients admitted to the ICU in order to decrease the prevalence and spread of these infections in ICU settings. Therefore, the aim of this study was to analyze the characteristics and outcomes of patients with infections caused by MDR *A. baumannii* in a tertiary care teaching hospital and to determine the risk factors for in-hospital mortality.

## 2. Materials and Methods

### 2.1. Study Population and Bacterial Strains

This retrospective study was conducted at a 2213-bed tertiary care teaching hospital. All hospitalized patients with an *A. baumannii*-positive culture in 2016, 2017, 2020, and 2021 were recruited in this study. If *A. baumannii* was isolated from two or more sources of the same patient, only one isolate was included for analysis. In cases with different susceptibility results, the isolate showing the highest level of resistance was included. Wounds, biopsy, bronchial secretions, sputum, blood, pus, abdominal fluid, pleural fluid, urine, and cerebrospinal fluid (CSF) of hospitalized patients were the sources of specimen collection.

All patients were older than 18 years. The following characteristics from all patients were collected: demographics, chronic comorbid diseases illnesses (diabetes mellitus, heart disease, renal and hepatic failure, and cancer), surgery, source of infection, laboratory and clinical findings on the day of *A. baumannii* cultivation, hospitalization ward (ICU, medical wards, or surgical wards) at the beginning of the infection, duration of IMV before *A. baumannii* infection, and radiological findings (a new lung infiltrate or consolidation in chest X-ray) in the presence of *A. baumannii* growth in the cultures of bronchial aspirate or sputum. Sputum was accepted in cases without orotracheal intubation or tracheostomy if the culture was pure and the sample was representative of lower airways (absence of epithelial cells and ≥25 polymorphonuclear neutrophils per microscopic field).

Length of hospital and ICU stay before and after the development of *A. baumannii* infection, antimicrobial agents prescribed during hospitalization before *A. baumannii* infection, adjusted antibiotic therapy (when data about the susceptibility of the isolate were obtained), and cause of death were recorded.

*A. baumannii* detected in the culture was considered colonization when clinical and laboratory data did not indicate any infection. In such cases, no treatment was prescribed. For example, *A. baumannii* colonization in the lower respiratory tract is considered when sputum or the culture from bronchial secretions is positive for *A. baumannii*, but there are no symptoms or signs of respiratory tract infection, and chest radiography shows no signs of pulmonary infiltrate [21,22]. Monotherapy was defined as a treatment with a single antibiotic agent active against *A. baumannii* in vitro, while combination therapy was defined as a treatment with 2 or more antibiotics.

HAI was defined based on the surveillance definition of the Centers for Disease Control and Prevention (CDC)/National Healthcare Safety Network (NHSN) [23]. The study was approved by Kaunas Regional Biomedical Research Ethics Committee (No. BE10-0016, dated 28 December 2021). The need for written consent was waived due to the retrospective nature of the study. This study was a part of the larger study conducted in the tertiary care teaching hospital [24].

### 2.2. Antimicrobial Susceptibility Testing

Antimicrobial susceptibility testing was performed by a disk diffusion method on Müller–Hinton agar (MH II according to EUCAST, Graso Biotech Microbiology Systems, Owidz, Poland). All inoculated plates were incubated for 16–20 h at 35 °C ± 1 °C in an ambient air incubator after inoculation with organisms and placement of disks. All the strains were tested for sensitivity to ceftazidime, cefepime, gentamicin, amikacin, ciprofloxacin, ampicillin/sulbactam, piperacillin/tazobactam, cefoperazone/sulbactam, imipenem, meropenem, doxycycline, tigecycline, tetracycline, sulfamethoxazole/trimethoprim, and colistin by using BD BBL™ Sensi-Disc™ antimicrobial susceptibility test disks (Becton Dickinson and Company, Franklin Lakes, NJ, USA). The diameter of the inhibition zone was measured in millimeters using a ruler. Inhibition zone diameters were interpreted according to the European Committee on Antimicrobial Susceptibility Testing (EUCAST) recommendations [25] and Clinical and Laboratory Standards Institute [26,27].

All isolated and identified *A. baumannii* strains were frozen at −80 °C. Isolates were defined as MDR if they were resistant to at least one antimicrobial agent from three or more classes of antimicrobials: penicillins (ampicillin/sulbactam, piperacillin/tazobactam) or cephalosporins (ceftazidime, cefepime), fluoroquinolones (ciprofloxacin), and aminoglycosides (amikacin, tobramycin, gentamicin).

Carbapenemase-producing isolates were detected phenotypically by a four-combination disk test (CDT), where antibiotic disks containing a beta-lactamase inhibitor (such as clavulanic acid or cloxacillin) show a significant increase in the inhibition zone compared to the antibiotic (cefotaxime) alone, which was detected during the production of extended-spectrum β-lactamases (ESBLs) and AmpC (Abtek Biologicals, Liverpool, UK, in 2016 and 2017 REF 21-ESBLAMPC; Liofilchem^®^, Roseto degli Abruzzi, Italy, REF 99008 in 2021 and 2022). Carbapenemase-producing isolates were detected phenotypically by a combination meropenem disk test. A bacterial *A. baumannii* suspension (0.5 McFarland) was inoculated, and four meropenem disks with or without various inhibitors (such as EDTA or phenylboronic acid) were placed. The results of the combination meropenem disk test were interpreted based on the comparison between the inhibition zones of the four meropenem disks. This test offers an advantage as it discriminates between carbapenem-susceptible, KPC-producing, metallo-β-lactamase (MBL)-producing, and double carbapenemase-producing bacteria [24,28].

### 2.3. Antimicrobial Treatment Decision and Appropriateness of Therapy

Treatment with antibiotics was administered based on the recommendations by infectious disease consultants (clinical pharmacologists and microbiologists). Colistin was prescribed as monotherapy or in combination with other antibiotics (meropenem or cefoperazone/sulbactam) following the clinical solution. Intravenous antibiotics were administered as follows: colistin (a loading dose of 9 million international units; after 12 h, a maintenance dose of 3 million international units every 12 h); ampicillin/sulbactam (combination of 2 g ampicillin + 1 g sulbactam every 6 h); tigecycline (a loading dose of 100–200 mg followed by 50–100 mg every 12 h); meropenem (1–2 g every 8 h in extended infusion); cefoperazone/sulbactam (combination of 1 g sulbactam + 1 g cefoperazone every 12 h); and ciprofloxacin (in case of urinary tract infection, 0.4 g every 8 h). The doses of all antibiotics were corrected considering kidney function parameters according to the manufacturer’s recommendations. Treatment was continued or adjusted based on the final results of isolate susceptibility and the dynamics of inflammatory markers.

### 2.4. Statistical Analysis

Statistical analysis performed using the Statistical Package for the Social Sciences (SPSS 29.0). The Kolmogorov–Smirnov test was employed to determine how continuous data were distributed. If continuous data were normally distributed, they were expressed as means with standard deviations (SD); if non-normally, they were expressed as medians with interquartile ranges (IQR). More than two independent groups of non-normally distributed data were compared with the Kruskal–Wallis test using the Dunn post hoc test for pair-wise comparison. Categorical data were expressed as numbers with percentages. Associations between categorical data were evaluated by a chi-square criterion; in cases when the frequency in at least one table cell was small, the Fisher exact test was used. Logistic regression analysis was performed to determine the risk factors associated with in-hospital mortality. All variables with *p* < 0.05 in the univariate analysis were included in a multivariate model. Odds ratios (ORs) and their 95% confidence intervals (95% CI) were calculated. Receiver operating characteristic (ROC) analysis was used to determine the area under the curve (AUC) and the cutoff values of the age and length of hospital stay after the detection of *A. baumannii* infection to predict in-hospital mortality. The level of significance was set at *p* < 0.05.

## 3. Results

A total of 196 isolates were collected: 60 (30.6%) from women and 136 (69.4%) from men, with a mean age of 61.7 ± 16.6 (range, 52–74) years. The mean length of hospital stay was 25.24 ± 30 (IQR, 7–34) days, and the mean length of ICU stay was 19 ± 46 (IQR, 3–20) days. The highest number of *A. baumannii* strains were isolated from patients hospitalized in ICU (43.4%, *n* = 85), followed by surgical wards (31.1%, *n* = 61) and medical wards (25.5%, *n* = 50). More than three-fourths (76.5%, *n* = 150) of the patients had at least one comorbid disease. Cardiac diseases accounted for 62.2% (*n* = 122) of all comorbid diseases; cancer, for 27.5% (*n* = 54); type 2 diabetes mellitus, for 18.0% (*n* = 35); and type 1 diabetes mellitus, for 2.1% (*n* = 4).

Of the 196 episodes of infection, respiratory infections caused by *A. baumannii* made up 66.3% (*n* = 130); skin and soft tissue infections plus surgical wound infections, 8.7% (*n* = 17); bacteremia, 6.6% (*n* = 13); gastrointestinal tract infections, 5.6% (*n* = 11); urinary tract infections, 4.1% (*n* = 8); and infections of other locations, 1.0% (*n* = 2). As many as 7.7% (*n* = 15) of cases were considered contamination.

Along with *A. baumannii*, one co-pathogen was found in 94 (47.9%) patients and two or more co-pathogens, in 102 (52.0%) patients. In cases of co-infection, *Klebsiella pneumoniae* and *Pseudomonas aeruginosa* were isolated most often with *A. baumannii.*

Before the diagnosis of *A. baumannii* infection, patients were treated with 2 antibacterial drugs on average (SD, 1; range, 0–5). Cephalosporins were the most frequently prescribed drugs (*n* = 147, 75.0%) followed by penicillin + *β*-lactamase inhibitors (BLI) (*n* = 112, 57.1%), carbapenems (*n* = 76, 38.8%), antifungals (*n* = 33, 16.8%), and quinolones (*n* = 25, 12.8%). The majority of the patients (81.6%, *n* = 160) were subjected to IMV lasting for 7 days on average (SD, 7; range, 1–51).

*A. baumannii* strains producing three types of *β*-lactamases were more frequently isolated from females than males (77.8% vs. 22.2%, *p* = 0.006). Infections caused by *A. baumannii* strains producing two types of *β*-lactamases were significantly more often treated with combination therapy than infections caused by strains producing one type of *β*-lactamase (78.9% vs. 60.0%, *p* = 0.019). No significant associations were found between the type and number of *β*-lactamases and patients’ characteristics such as age, cause of hospitalization mechanical ventilation, length of stay before *A. baumannii* infection, markers of inflammation, chronic diseases, and antibiotic treatment before infection (Table 1).

**Figure 1:**
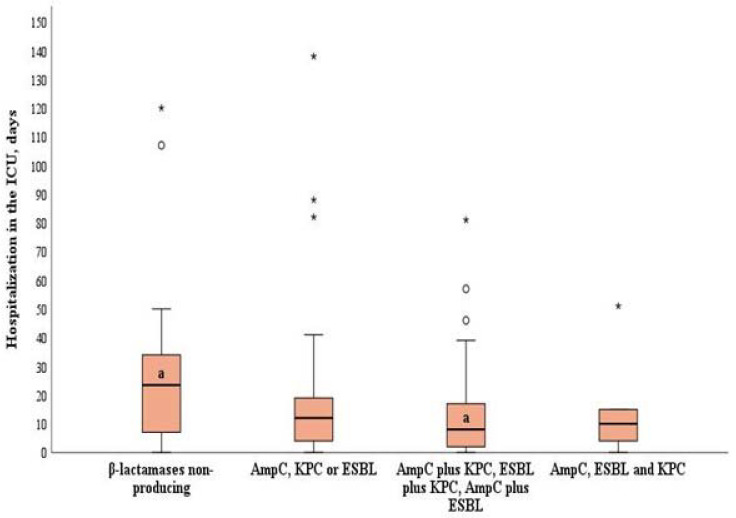
Length of stay in the ICU by the production of *β*-lactamases in *A. baumannii* strains. χ^2^ = 9.613, df = 3, *p* = 0.022 by the Kruskal–Wallis test (Dunn post hoc test for pair-wise comparison: *β*-lactamase non-producing strains vs. strains producing two types of *β*-lactamases, *p* = 0.016). Error bars indicate the range of distribution; the box, the interquartile range; the horizontal line, the median value; and the asterisks and circles, outliers.

Patients with *A. baumannii* strains producing two different types of *β*-lactamases (AmpC plus KPC, AmpC plus ESBL, or ESBL plus KPC) stayed significantly shorter at the ICU compared to patients with *A. baumannii* strains not producing *β*-lactamases (median of 9, IQR 2–18, vs. median of 26, IQR 7–38, *p* = 0.022) (Figure 1).

Despite there being no significant associations between the type of antibacterial treatment and *A. baumannii* strains producing different types of *β*-lactamases before the detection of *A. baumannii* infection (*p* > 0.05) (Table 1), possible associations were evaluated after the detection of *A. baumannii* infection.

Of the 196 patients with *A. baumannii* infection, 73 received combination therapy with colistin and BLI (37.2%), 28 received monotherapy with BLI (14.3%), 16 continued empirical treatment (8.2%), 16 received combination therapy with colistin and carbapenems (8.2%), and other combinations were administered in 12 patients (6.1%). Death occurred in 34 patients (17.3%) before treatment could be administered, and 17 cases (8.7%) of *A. baumannii* infection were considered as colonization. Monotherapy was administered to 23.5% of patients to treat *A. baumannii* infection and combination therapy, in 59.2%.

Patients with infections caused by *A. baumannii* producing one type of *β*-lactamase were significantly more frequently treated with the combination of colistin and carbapenem, as well as other combinations, than those with infections caused by *A. baumannii* producing two types of *β*-lactamases (57.1% and 63.6% vs. 35.7% and 36.4%, *p* = 0.015 and *p* = 0.017, respectively). The combination of colistin with BLI was administered significantly more frequently to treat infections caused by *A. baumannii* producing two types of *β*-lactamases than infections caused by *A. baumannii* producing one type of *β*-lactamase (83.8% vs. 11.8%, *p* < 0.001). The detailed information is shown in Table 2.

Of the 196 patients diagnosed with *A. baumannii* infection, 58.7% (*n* = 115) died and 41.3% (*n* = 81) survived. Demographic and clinical characteristics of survivors and non-survivors are shown in Table 3. Compared to survivors, non-survivors were significantly older (55.9, SD 17.9, vs. 65.9, SD 14.2, years), were more likely to have chronic cardiovascular and respiratory diseases (49.4% vs. 71.3% and 65.4 vs. 91.3%, respectively), were more likely to be hospitalized due to surgery (48.2% vs. 51.8%), and were more likely to be ventilated (69.1% vs. 90.4%). The median length of stay in hospital after *A. baumannii* infection for non-survivors was 6 days (IQR, 1–21) compared to 21 days (12.5–41.5) for those who survived.

Among the non-survivors, death occurred significantly more frequently in those who were treated with combination therapy than those who were treated with monotherapy (84.4% vs. 15.6%) and in those who were not co-infected with other bacteria than those who had co-infections (56.5% vs. 43.5%).

Receiver operating characteristic (ROC) curve analysis was performed to assess the ability of age and the duration of effective antibiotic treatment to predict in-hospital mortality in patients with drug-resistant *A. baumannii*. An age of >58 years had an AUC of 0.66 (95% (CI), 0.59–0.72), sensitivity of 71.3%, and specificity of 54.3% (*p* < 0.001), and a duration of effective antibiotic treatments of ≤6 days had an AUC of 0.72 (95% CI, 0.65–0.78), sensitivity of 66.09%, and specificity of 82.72% (*p* < 0.001).

Binary logistic regression analysis revealed the following significant risk factors for in-hospital mortality: a duration of effective antibiotic treatment for ≤6 days was associated with a 6.92-fold greater risk of in-hospital mortality (95% CI, 2.87–16.66); IMV, with a 5.58-fold greater risk of in-hospital mortality (95% CI, 1.93–16.17); age of >58 years, with a 4.99-fold greater risk (95% CI, 2.19–11.33); combination therapy, with a 3.62-fold greater risk (95% CI, 1.35–8.36); and no co-infection, with a 2.35-fold greater risk (95% CI, 1.07–5.14) (Table 4).

## 4. Discussion

*A. baumannii* is one of the most common opportunistic agents causing HAIs, especially in ICU settings [29,30]. The global estimated incidence of *A. baumannii* infections is approximately one million cases per year, and due to resistance and the lack of treatment options, hospital-acquired *A. baumannii* infections are associated with high mortality, especially in critically ill patients [31,32]. This study aimed to analyze the characteristics of patients with infections caused by MDR *A. baumannii* and the clinical outcomes of these patients, as well as to identify the risk factors contributing to infection-related in-hospital mortality in a tertiary care teaching hospital.

In our study, the incidence of *A. baumannii* infections in the ICU setting was lower than that reported in the study by Calò et al. [33] (43.4% vs. 52.5%), but was higher than that in the study by Abarca-Coloma et al. [10], in which 21.7% of the detected infections occurred in the ICU. This difference in incidence rates may be explained by the use of infection control measures, which should be followed according to the CDC guidelines for infection control in healthcare [34]. The study by Uwingabiye et al. [35] showed that patients who developed ICU-acquired *A. baumannii* infections had a median ICU length of 18 (IQR: 10–26) days; in our study, the median length of ICU stay was 10 (IQR, 3–20) days. Appaneal et al. [36] reported that a length of stay >10 days was more common among those with MDR *A. baumannii* versus non-MDR *A. baumannii*, suggesting that ICU-acquired *A. baumannii* infections are due to prolonged ICU stays. Unnecessary hospitalization days may increase the rate of hospital-acquired complications and economic burden [37]. Long stays in the ICU and the use of medical devices are necessary for the treatment of critically ill patients in modern medicine, but their presence is associated with the risk of infection. Previous studies have identified IMV as a possible risk factor for ventilator-associated pneumonia (VAP) and bacteremia [38,39]. The study by Abarca-Coloma et al. [20] showed that the most important factor associated with mortality was IMV and the consequent VAP, followed by hemodialysis and a history of chronic renal failure. In our study, we also found that exposure to IMV was associated with mortality in patients with *A. baumannii* infections. This explains why *A. baumannii* isolates were most commonly found in the respiratory tract of our patients (66.3%), and this is consistent with the findings of the study by Hafiz et al. [40], who found that respiratory infections caused by *A. baumannii* accounted for 63% of all *A. baumannii*-related infections. According to the findings of other studiesy investigating the prevalence of *A. baumannii* in the samples collected from different sources, including blood, the respiratory tract, and urine, the lower respiratory tract also represented the most common source of infection (67%) [41].

In our study, of the 196 patients, 17 (8.7%) were colonized, whereas infection by *A. baumannii* was diagnosed in 179 (91.3%) individuals. In another study, a high proportion of patients (60%) was colonized by *A. baumannii* [42]. Antibiotic exposure is one of the most frequently reported risk factors for MDR *A. baumannii* colonization or infection, and the use of carbapenems, third-generation cephalosporins, and *β*-lactams has been reported [43,44,45]. Antibiotic therapy facilitates the emergence of new resistant mutants or the proliferation of antibiotic-resistant *A. baumannii* by exerting selective pressure, which greatly limits the treatment options for infections caused by *A. baumannii*, posing a significant challenge [46].

Carbapenemases produced by *A. baumannii* hydrolyze all beta-lactam antibiotics, including carbapenems, which poses a serious problem with limited therapeutic options [47]. As in our study, patients with infections caused by *A. baumannii* producing carbapenemases and AmpC or ESBLs were treated with a combination therapy consisting of colistin with cefoperazone/sulbactam. Meanwhile, patients with infections caused by *A. baumannii* producing one β-lactamase (AmpC or KPC or ESBL) were more commonly treated with a combination therapy consisting of colistin with carbapenems or other combinations. This can be explained by the fact that, in the case of *A. baumannii* producing ESBL and AmpC β-lactamases, sensitivity to carbapenems is retained, and such infections are most commonly treated with carbapenems [48]. Carbapenems resistant to hydrolysis can be inactivated by plasmid AmpCs in combination with ESBLs, and this makes Gram-negative bacteria insusceptible to carbapenem agents [49].

In our study, monotherapy to treat *A. baumannii* infection was administered in 23.5% of patients and combination therapy was administered in 59.2%. In the study by López-Cortés et al. [50], 101 patients with sepsis caused by multidrug-resistant *A. baumannii* were evaluated in 28 Spanish hospitals. The study reported that 67.3% of patients received monotherapy, while 32.7% received combination therapy. In the study by Park et al., 44.6% of patients with *A. baumannii* infections were treated with monotherapy and 55.4% were treated with combination therapy [51] given at a similar frequency, as in our study. Combination antibiotic therapy is prescribed to treat more serious infections caused by MDR *A. baumannii* and polymicrobial infections [52]. Univariate analysis showed that combination therapy was significantly associated with higher patient in-hospital mortality compared to patients receiving monotherapy for MDR *A. baumannii* infections. The Infectious Diseases Society of America (IDSA) guidelines indicated cefiderocol as part of combination antibiotic regimens in carbapenem-resistant *A. baumannii* infections refractory to other antibiotics or in case of intolerance to other agents [11]. A conditional recommendation against cefiderocol for the treatment of infections caused by carbapenem-resistant *A. baumannii* has been conversely stated by the ECCMID [10].

In our study, in-hospital mortality among patients with *A. baumannii*-caused infection was 58.7%, which is comparable with mortality in other study [53]. The mortality rate among patients with *A. baumannii*-caused infections across the world ranges between 26% and 60% [53,54,55]. The high mortality rate can be attributed to several factors, including comorbid medical conditions, antimicrobial resistance, and inappropriate treatment [56]. Therefore, timely diagnosis in patients with risk factors in critical settings and the consequent immediate treatment of *A. baumannii* infections could contribute to reducing mortality rates [20]. Numerous studies analyzing the risk factors for mortality among patients with *A. baumannii* infections have reported that many comorbidities such as chronic liver, cardiovascular, and renal diseases, as well as more severe diseases, i.e., septic shock, or higher APACHE II or Pitt bacteremia scores, are associated with increased mortality rates [36,55,57]. The study by Abarca-Coloma et al. [20] showed that the risk factors for mortality in patients with hospital-acquired carbapenem-resistant *A. baumannii* infections were IMV exposure, hemodialysis, and a history of chronic renal failure. Our study found that combination therapy of *A. baumannii* infection, the duration of effective antibiotic treatment for ≤6 days, an age of >58 years, and no co-infection were significantly associated with a greater risk of in-hospital mortality. However, we did not observe any significant association between the production of different types and numbers of *β*-lactamases and clinical patients’ outcomes.

Some limitations of this study must be acknowledged. First, as it was a single-center study, the results of this study may not be generalizable to other hospitals or healthcare settings; therefore, there is a possibility that the findings may not accurately reflect the characteristics of the broader population of patients with MDR *A. baumannii* infections. Second, *A. baumannii* strains were not tested in 2018, 2019, and 2020, which means that some strains may be associated with potentially higher drug resistance. Third, as the study was conducted retrospectively, there is a chance for biases to be present in the selection of patients and data collection. Finally, only phenotypic analysis was carried out for isolates; therefore, not all potential beta-lactamase producers could be identified. Due to all the above-mentioned limitations, the results of our study should be interpreted with caution and cannot be overgeneralized. For more accurate results, larger multicenter prospective studies in different countries and healthcare institutions would be relevant. Such multicenter prospective studies would provide a better understanding of the role of MDR *A. baumannii* as a causative pathogen in in-hospital mortality.

## 5. Conclusions

MDR *A. baumannii* infections pose a significant threat to human health, not only due to multidrug resistance but also due to high mortality. The mortality rate of patients with MDR *A. baumannii* infection was high and was associated with age, invasive mechanical ventilation, the duration of effective antibiotic treatment, no co-infection, and combination therapy. Therefore, it is of utmost importance to reduce the prevalence of MDR *A. baumannii* infections in healthcare facilities by applying preventive measures and to administer timely effective treatment once *A. baumannii* infection is detected.

## Figures and Tables

**Table 1:** Comparison of demographic characteristics, admission type, and underlying comorbidities and infection-related details between the pathogen *A. baumannii* producing different types and numbers of *β*-lactamases.

Variable	*A. baumannii* Strains Producing Different Types and Numbers of *β*-Lactamases			*p*
	One Type * (*n* = 53)	Two Different Types ** (*n* = 115)	All Three Types *** (*n* = 9)	
Gender, *n* (%)				
Female	16 (30.2)	31 (27.0)	7 (77.8) ^a^	
Male	37 (69.8)	84 (73.0)	2 (22.2) ^b^	0.006 ^a vs. b^
Age, mean (SD), years	61.6 (17.5)	62 (16.1)	63.6 (16.8)	0.949
Cause of hospitalization, *n* (%)				
Surgery	40 (75.5)	83 (72.2)	6 (66.7)	0.825
Trauma	7 (13.2)	7 (6.1)	0 (0)	0.188
Comorbid disease, *n* (%)	41 (77.4)	87 (75.7)	7 (77.8)	0.965
Antibiotic treatment before infection (class), *n* (%)				
Cephalosporin	41 (77.4)	86 (74.8)	6 (66.7)	0.781
Penicillin + BLI	31 (58.5)	69 (60.0)	4 (44.4)	0.658
Carbapenem	22 (41.5)	39 (33.9)	5 (55.6)	0.325
Antifungal	8 (15.1)	22 (19.1)	0 (0)	0.308
Quinolone	9 (17.0)	10 (8.7)	0 (0)	0.154
Antibiotic treatment after infection, *n* (%)				
Monotherapy	18 (40.0) ^a^	20 (21.1) ^b^	1 (14.3)	0.019 ^a vs. b^
Combination therapy	27 (60.0) ^a^	75 (78.9) ^b^	6 (85.7)	0.019 ^a vs. b^
Length of stay before *A. baumannii* infection, days	17 (10–30)	13 (6–21)	14 (8–33)	0.353
Length of ICU stay before *A. baumannii* infection, days	6 (2–11)	5 (2–10)	4 (2–13)	0.908
IMV duration before *A. baumannii* infection, days	5 (2–10)	4 (2–9)	5 (2–19)	0.497
Inflammatory markers on *A. baumannii* infection onset				
WBC, ×10^9^/L	12.3 (7.3–16.2)	11.2 (7.8–15.7)	12.5 (4.9–16.6)	0.988
CRP, mg/L	126.5 (76.0–250.3)	108 (88.8–272.6)	236.9 (86.8–295.9)	0.294

Values are median (interquartile range) unless stated otherwise. * AmpC, KPC, or ESBL, ** AmpC Plus KPC, AmpC Plus ESBL, or ESBL Plus KPC, *** AmpC, KPC, and ESBL. BLI, *β*-lactamase inhibitors; ICU, intensive care unit; IMV, invasive mechanical ventilation; WBC, white blood cells; CRP, C-reactive protein; ^a vs. b^ indicates a significant difference between the groups labelled with superscript letters a and b.

**Table 2:** Associations between *A. baumannii* strains producing different numbers and types of *β*-lactamases and different treatment regimens after the detection of *A. baumannii* infection.

Treatment Groups	*A. baumannii* Strains Producing Different Types and Numbers of *β*-Lactamases		
	One Type * (*n* = 53)	Two Different Types ** (*n* = 115)	All Three Types *** (*n* = 9)
Did not receive an effective antibiotic (*n* = 46)	16 (34.8)	27 (58.7)	3 (6.7)
Continuation of empirical treatment ^&^ (*n* = 15)	3 (20.0)	10 (66.7)	2 (13.3)
Monotherapy with BLI ^†^ (*n* = 23)	11 (47.8)	12 (52.2)	0 (0.0)
Combination of colistin with BLI ^√^ (*n* = 68)	8 (11.8) ^a^	57 (83.8) ^b^	3 (4.4)
Combination of colistin and imipenem or meropenem (*n* = 14)	8 (57.1) ^a^	5 (35.7) ^b^	1 (7.1)
Other combinations ^#^ (*n* = 12)	7 (63.6) ^a^	4 (36.4) ^b^	0 (0.0)

χ^2^ = 30.36, df = 10, *p* < 0.005. Significantly different ^a^ vs. ^b^. * AmpC, KPC, or ESBL, ** AmpC Plus KPC, AmpC Plus ESBL, or ESBL Plus KPC, *** AmpC, KPC, and ESBL. BLI, *β*-lactamase inhibitors; ^&^ carbapenems (imipenem or meropenem), ampicillin/sulbactam, cefoperazone/sulbactam; ^†^ ampicillin/sulbactam or cefoperazone/sulbactam; ^√^ cefoperazone/sulbactam; ^#^ ciprofloxacin, doxycycline, or colistin + doxycycline.

**Table 3:** Demographic and clinical characteristics of survivors and non-survivors.

Variable	Survivors (*n* = 81)	Non-Survivors (*n* = 115)	*p*
Gender, *n* (%)			
Female	23 (28.4)	37 (32.2)	0.572
Male	58 (71.6)	78 (67.8)	
Age, mean (SD), years	55.9 (17.9)	65.9 (14.2)	<0.001
Comorbid diseases, *n* (%)			
Cardiovascular	40 (49.4)	82 (71.3)	0.002
Respiratory	53 (65.4)	105 (91.3)	<0.001
Renal	7 (8.6)	16 (13.9)	0.259
Liver	4 (5.1)	15 (13.0)	0.085
Diabetes mellitus	14 (17.3)	25 (22.2)	0.591
Cancer	20 (24.7)	34 (29.6)	0.452
At least one comorbid disease	55 (67.9)	95 (82.6)	0.017
Cause of hospitalization, *n* (%)			
Surgery	68 (84.0)	73 (63.5)	0.002
Trauma	10 (12.3)	5 (4.3)	0.038
IMV	56 (69.1)	104 (90.4)	< 0.001
Length of hospital stay before ICU, days	10 (1–19.7)	10 (4–21)	0.146
Length of ICU stay before *A. baumannii* infection, days	5 (1–12)	5 (2–10)	0.450
Length of hospital stay after *A. baumannii* infection, days	21 (12.5–41.5)	6 (1–21)	<0.001
Duration of IMV before *A. baumannii* infection, days	3.0 (2.3–10)	3.5 (2–9)	0.212
Duration of antibiotic treatment before *A. baumannii* infection, median (IQR), days	15.0 (8–24.5)	14.0 (8–23.0)	0.649
Treatment, *n* (%)			
Did not receive effective antibiotic treatment	14 (27.5)	37 (72.5)	<0.019
Received effective antibiotic treatment	67 (46.2)	78 (53.8)	
Antibiotic treatment after infection, *n* (%)			
Monotherapy	33 (40.7)	13 (16.0)	0.045
Combination therapy	48 (59.3)	68 (84.0)	
Duration of effective antibiotic treatment for *A. baumannii* infection, median (IQR), days	10.0 (7–14)	3 (0–8)	<0.001
Co-infection with other bacteria, *n* (%)			
No	29 (35.8)	65 (56.5)	<0.001
Yes	52 (64.2)	50 (43.5)	
*A. baumannii* strains producing different types and numbers of *β*-lactamases, *n* (%)			
One type *	26 (32.1)	27 (23.5)	0.407
Two different types **	46 (56.8)	69 (60.0)	
All three types ***	2 (2.5)	7 (6.1)	

Values are median (interquartile range) unless stated otherwise. ICU, intensive care unit; IMV, invasive mechanical ventilation. * AmpC, KPC, or ESBL, ** AmpC Plus KPC, AmpC Plus ESBL, or ESBL Plus KPC, *** AmpC, KPC, and ESBL.

**Table 4:** Risk factors for in-hospital mortality among patients with MDR *A. baumannii* infection.

Risk Factor	Beta Coefficient	SE	Wald	OR (95% CI)	*p*
Effective antibiotic treatment for ≤6 days	1.93	0.45	18.59	6.92 (2.87–16.66)	<0.001
IMV	1.72	0.54	10.05	5.58 (1.93–16.17)	0.002
Age of >58 years	1.61	0.42	14.75	4.99 (2.19–11.33)	<0.001
Combination therapy	1.21	0.47	6.81	3.62 (1.35–8.36)	0.009
No co-infection	0.85	0.40	4.55	2.35 (1.07–5.14)	0.033

IMV, invasive mechanical ventilation; SE, standard error; OR, odds ratio, CI, confidence interval.

## Data Availability

The data presented in this study are available on request from the corresponding author.
